# Diagnostic yield of diffusion-weighted brain MR imaging in patients with cognitive impairment: Large cohort study with 3,298 patients

**DOI:** 10.1371/journal.pone.0274795

**Published:** 2022-09-22

**Authors:** Minjae Kim, Sang Yeong Kim, Chong Hyun Suh, Woo Hyun Shim, Jae-Hong Lee, Jeffrey P. Guenette, Raymond Y. Huang, Sang Joon Kim

**Affiliations:** 1 Department of Radiology and Research Institute of Radiology, Asan Medical Center, University of Ulsan College of Medicine, Seoul, Republic of Korea; 2 University of Ulsan College of Medicine, Seoul, Republic of Korea; 3 Department of Neurology, Asan Medical Center, University of Ulsan College of Medicine, Seoul, Republic of Korea; 4 Division of Neuroradiology, Brigham and Women’s Hospital, Dana-Farber Cancer Institute, Harvard Medical School, Boston, Massachusetts, United States of America; National Institutes of Health, UNITED STATES

## Abstract

**Objective:**

There is a paucity of large cohort-based evidence regarding the need and added value of diffusion-weighted imaging (DWI) in patients attending outpatient clinic for cognitive impairment. We aimed to evaluate the diagnostic yield of DWI in patients attending outpatient clinic for cognitive impairment.

**Materials and methods:**

This retrospective, observational, single-institution study included 3,298 consecutive patients (mean age ± SD, 71 years ± 10; 1,976 women) attending outpatient clinic for cognitive impairment with clinical dementia rating ≥ 0.5 who underwent brain MRI with DWI from January 2010 to February 2020. Diagnostic yield was defined as the proportion of patients in whom DWI supported the diagnosis that underlies cognitive impairment among all patients. Subgroup analyses were performed by age group and sex, and the Chi-square test was performed to compare the diagnostic yields between groups.

**Results:**

The overall diagnostic yield of DWI in patients with cognitive impairment was 3.2% (106/3,298; 95% CI, 2.6–3.9%). The diagnostic yield was 2.5% (83/3,298) for acute or subacute infarct, which included recent small subcortical infarct for which the diagnostic yield was 1.6% (54/3,298). The diagnostic yield was 0.33% (11/3,298) for Creutzfeldt-Jakob disease (CJD), 0.15% (5/3,298) for transient global amnesia (TGA), 0.12% (4/3,298) for encephalitis and 0.09% (3/3,298) for lymphoma. There was a trend towards a higher diagnostic yield in the older age group with age ≥ 70 years old (3.6% vs 2.6%, *P* = .12). There was an incremental increase in the diagnostic yield from the age group 60–69 years (2.6%; 20/773) to 90–99 years (8.0%; 2/25).

**Conclusion:**

Despite its low overall diagnostic yield, DWI supported the diagnosis of acute or subacute infarct, CJD, TGA, encephalitis and lymphoma that underlie cognitive impairment, and there was a trend towards a higher diagnostic yield in the older age group.

## Introduction

Current guidelines recommend structural imaging for the evaluation of patients presenting with cognitive impairment [1], and MRI is the modality of choice revealing a spectrum of changes due to vascular pathologies and white matter diseases in the brain [2]. The American College of Radiology (ACR) Appropriateness Criteria [3], the UK National Institute of Health and Care Excellence [4], and the European Federation of Neurological Societies [5,6] recommend structural imaging for the purpose of excluding treatable causes of dementia and evaluating the pattern of atrophy. The minimum sequences recommended to evaluate a patient with cognitive impairment include T2-weighted images and FLAIR images for assessing the degree of vascular changes and high resolution T1-weighted images for evaluating anatomy and atrophy [2].

Diffusion-weighted imaging (DWI) may be considered as a standard and fast sequence that is routinely performed for cognitive impairment. This was supported by the result of the recent survey by the European Society of Neurology which reported that DWI was included as a part of MR imaging protocol for dementia in 91% of institutions [7]. In the European Federation of the Neurological Societies guidelines, DWI was recommended as a good practice point to identify recent infarctions and changes related to Creutzfeldt-Jakob disease (CJD) [8]. However, there is a paucity of data-based evidence to support the need and added value of DWI in patients with cognitive impairment in a large cohort study. While conventional images such as T2-weighted images and FLAIR images may or may not show hyperintensities, DWI provides unique information that the conventional images may not be able to offer by demonstrating diffusion restriction. This may play an important role in formulating differential diagnoses and supporting diagnoses of important causes of cognitive impairment by showing cytotoxic edema of acute/subacute infarction, increased cellularity of tumor or vacuolization of neutrophils in CJD. Understanding the diagnostic yield of DWI in a large cohort study may provide evidence for establishing and refining guidelines in the evaluation of patients with cognitive impairment.

While DWI may support the diagnosis of important causes of cognitive impairment, the need and added value of DWI in patients with cognitive impairment have not been established in a large cohort study. Therefore, the purpose of this study was to evaluate the diagnostic yield of DWI in patients attending outpatient clinic for cognitive impairment.

## Materials and methods

### Patients

This retrospective, observational, single-institution study was approved by the institutional review board of Asan Medical Center with a waiver of written informed consent. A search of the electronic medical record of a tertiary referral hospital was performed to identify patients attending outpatient clinic for cognitive impairment who underwent brain MRI with DWI from January 2010 to February 2020. Patients with Clinical Dementia Rating < 0.5 were excluded. Patients who met the following eligibility criteria were included: (a) patients presented to outpatient clinic at the study site for initial evaluation of alleged cognitive impairment with clinical dementia rating ≥ 0.5, (b) patients underwent brain MRI that included DWI as a part of evaluation for cognitive impairment within one month of presentation to the outpatient clinic.

### Brain MRI including DWI

All patients presenting to the outpatient clinic with cognitive impairment at the study site underwent identical protocol which included DWI. In addition to DWI, the protocol consisted of three-dimensional (3D) T1-weighted image, FLAIR image, T2-weighted image and susceptibility-weighted image. All patients underwent MRI with a 3.0-T system (Achieva or Ingenia; Philips Medical Systems, Best, The Netherlands) using an eight-channel sensitivity-encoding head coil. The DWI parameters remained unchanged during the study period and were as follows: repetition time/echo time, 10788/70 ms; diffusion gradient encoding, b = 0 and 1000 s/mm^2^; field of view, 23 x 23 cm; matrix, 240 × 240; slice thickness/gap, 2/0 mm and number of slices, 70. Apparent diffusion coefficient (ADC) images were calculated from b = 1000 and b = 0 s/mm^2^ on DWI.

### MRI analysis and outcomes

Reports of brain MRI with DWI for study patients were reviewed. There were structured reports used at the study site for brain MRI with subheadings for each sequence, and positive findings identified on each sequence were stated separately. Contents of the reports on the DWI subheading were screened, and reports identifying positive findings on DWI were regarded test positive. The images of all test positive brain MRI were reviewed in consensus of two board-certified neuroradiologists (M.K. and C.H.S. with 5 and 9 years of experience in neuroradiology) who were blinded to all other clinical and imaging information. In case of discrepancy, the images were reviewed by the most experienced neuroradiologist (S.J.K. with 35 years of experience in neuroradiology). Images with lesions showing high signal intensity on DWI accompanied by low ADC were regarded as true positive with the exception of recent or subacute infarcts in which pseudonormalization of ADC was seen. Reports with no positive findings on DWI were regarded as test negative. Two board-certified neuroradiologists (M.K. and C.H.S.) reviewed images of randomly selected 200 patients whose reports identified no positive findings on DWI.

Clinical characteristics of patients with positive findings on DWI were also collected including the Clinical Dementia Rating [9], the Korean version of Mini-Mental State Examination and the level of education. The presence of vascular risk factors including hypertension, hyperlipidemia, diabetes mellitus, history of smoking, history of ischemic heart disease and atrial fibrillation was collected [10]. Patients whose reports identified positive findings on DWI were followed up to determine the final clinical diagnosis and clinical course of diseases, which included acute or subacute infarct, transient global amnesia (TGA), CJD, encephalitis and lymphoma. Acute or subacute infarct was identified as hyperintensity on DWI and hypointensity or isointensity on apparent diffusion coefficient map [11]. Acute or subacute infarcts were classified as territorial and nonterritorial infarcts. Territorial infarcts were infarcts due to large-artery atherosclerosis [12]. Nonterritorial infarcts included recent small subcortical infarct defined as neuroimaging evidence of recent infarct in the territory of one perforating arteriole [13]. Patients suspected of CJD on DWI were followed up to determine if they met the diagnostic criteria for CJD. The diagnosis of CJD was made according to the diagnostic criteria by the Centers for Disease Control and Prevention [14]. The fate of recent small subcortical infarct was determined if follow up images were available. Patients suspected of TGA on DWI were followed up to confirm if their symptoms of cognitive impairment improved subsequently and whether they met the criteria for TGA [15]. Diagnosis of encephalitis was confirmed by the clinicoradiologic consensus supported by clinical history, laboratory and imaging findings [16,17]. Lymphoma was confirmed histopathologically on stereotactic biopsy unless contraindicated.

### Statistical analysis

The primary outcome was the diagnostic yield of DWI in patients presenting to outpatient clinic with cognitive impairment. Diagnostic yield was defined as the proportion of patients with true-positive cases among all patients (number of true-positive results divided by total number of patients) and exact 95% CIs were determined [18,19]. False referral rate was defined as the proportion of patients with false-positive findings on DWI among all patients (number of false-positive results divided by total number of patients) [18]. Subgroup analyses were performed by age group and sex. Patients were categorized into two age groups considering the mean age, and the diagnostic yield was compared between age groups and sex using the Chi-square test. Diagnostic yields were also stratified by age groups with 10-year intervals from age 50 to 99. Statistical analyses were conducted using MedCalc, version 19.1 (MedCalc Software, Mariakerke, Belgium). The level of statistical significance was defined as *P* < .05.

## Results

### Patient characteristics

**Fig 1:**
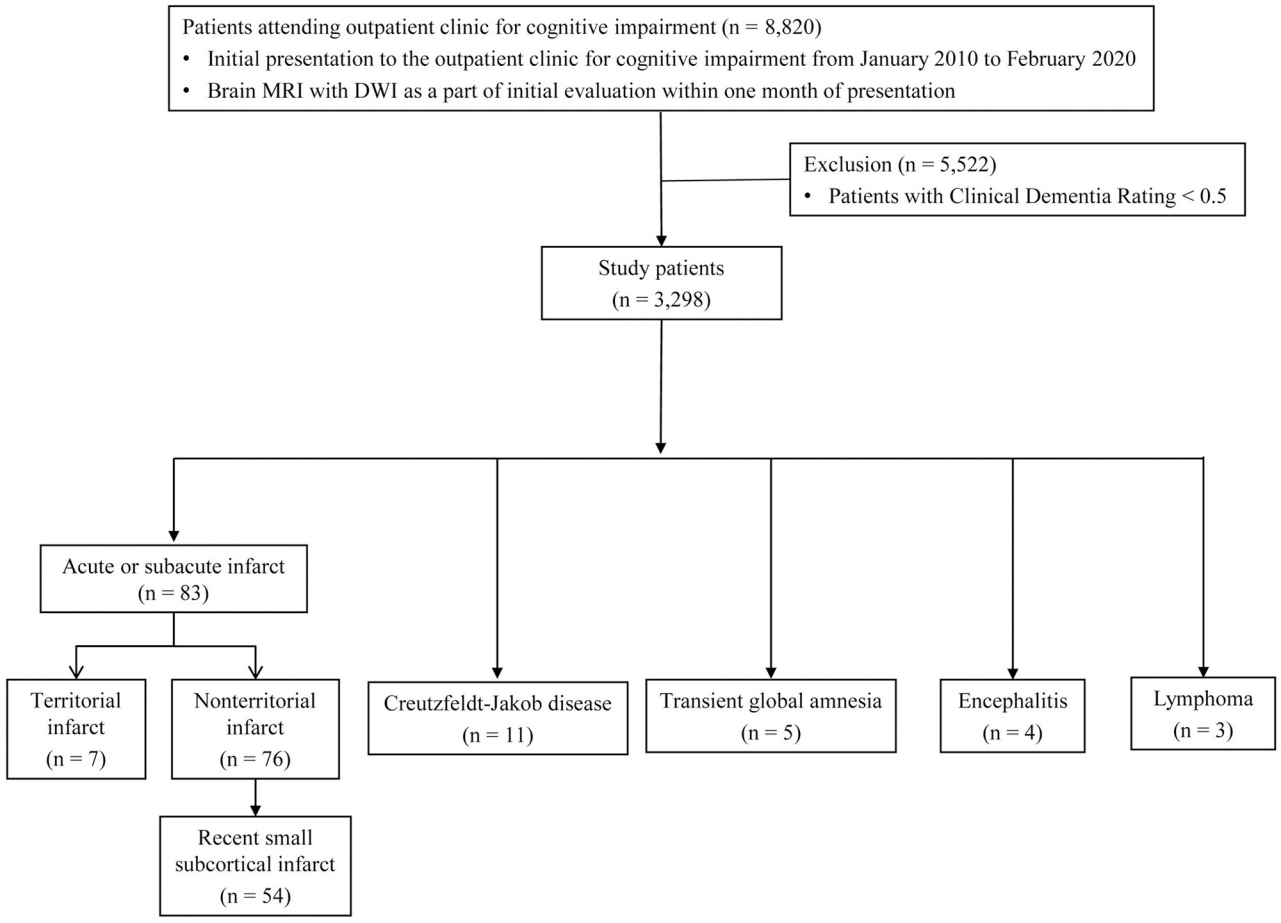
Patient flow diagram. DWI = diffusion-weighted imaging.

There were 8,820 patients attending outpatient clinic for cognitive impairment from January 2010 to February 2020. There were 5,522 patients with CDR < 0.5 who were excluded, and a total of 3,298 patients (mean age ± SD, 71 ± 10 years; 1,976 women) were included in the analysis. The patient flow diagram is presented in Fig 1.

### Diagnostic yield of DWI

The overall diagnostic yield and diagnostic yields per positive findings are presented in Table 1. Among 3,298 patients, there were positive findings on DWI in 106 patients. The overall diagnostic yield of DWI in patients presenting with cognitive impairment was 3.2% (106/3,298; 95% CI, 2.6–3.9%). There were no false positive cases, and the false referral rate was 0%. The mean MMSE for patients with positive findings on DWI was 21.1 (SD, 5.7).

**Table 1:** Diagnostic yield of diffusion-weighted imaging in patients with cognitive impairment and clinical characteristics of patients with positive findings.

	Diagnostic yield, % (proportion; 95% CI)	Mean age (range)	Women, no. (%)	K-MMSE (score ± SD)	Education (years ± SD)
Overall diagnostic yield	3.2 (106/3,298; 2.6–3.9%)	73 (24–90)	57 (54%)	21.1 ± 5.7	9.2 ± 5.8
Acute or subacute infarct	2.5 (83/3,298; 2.0–3.1%)	75 (24–90)	47 (57%)	21.3 ± 5.4	8.7 ± 5.8
Nonterritorial infarct	2.3 (76/3,298; 1.8–2.9%)	75 (24–90)	40 (53%)	21.7 ± 5.3	8.9 ± 5.6
Recent small subcortical infarct	1.6 (54/3,298; 1.2–2.1%)	75 (24–90)	31 (57%)	21.9 ± 4.7	8.6 ± 5.8
Territorial infarct	0.21 (7/3,298; 0.09–0.44%)	75 (71–85)	7 (100%)	19.4 ± 5.7	8.1 ± 7.5
Creutzfeldt-Jakob disease	0.33 (11/3,298; 0.09–0.27%)	62 (52–80)	5 (45%)	12.6 ± 7.2	9.0 ± 5.4
Transient global amnesia	0.15 (5/3,298; 0.05–0.35%)	66 (49–74)	2 (40%)	26.7 ± 2.1	12.5 ± 4.0
Encephalitis	0.12 (4/3,298; 0.03–0.31%)	67 (56–74)	2 (50%)	24.5 ± 4.8	12.5 ± 7.0
Lymphoma	0.09 (3/3,298; 0.02–0.27%)	72 (66–75)	1 (33%)	17.7 ± 2.5	10.8 ± 8.9

CI = confidence interval, K-MMSE = Korean version of Mini-Mental State Examination, SD = standard deviation.

The diagnostic yield was 2.5% (83/3,298) for acute or subacute infarct. This included recent small subcortical infarct for which the diagnostic yield of DWI was 1.6% (54/3,298). The diagnostic yield was 0.33% (11/3,298) for CJD, 0.15% (5/3,298) for TGA, 0.12% (4/3,298) for encephalitis and 0.09% (3/3,298) for lymphoma. There were no false negative cases when reviewing randomly selected 200 patients whose reports identified no positive findings on DWI. This consisted of patients with Alzheimer’s disease (32/200; 16%), mild cognitive impairment (129/200; 65%) and subjective memory impairment (39/200; 20%).

### MRI findings and outcome

**Fig 2:**
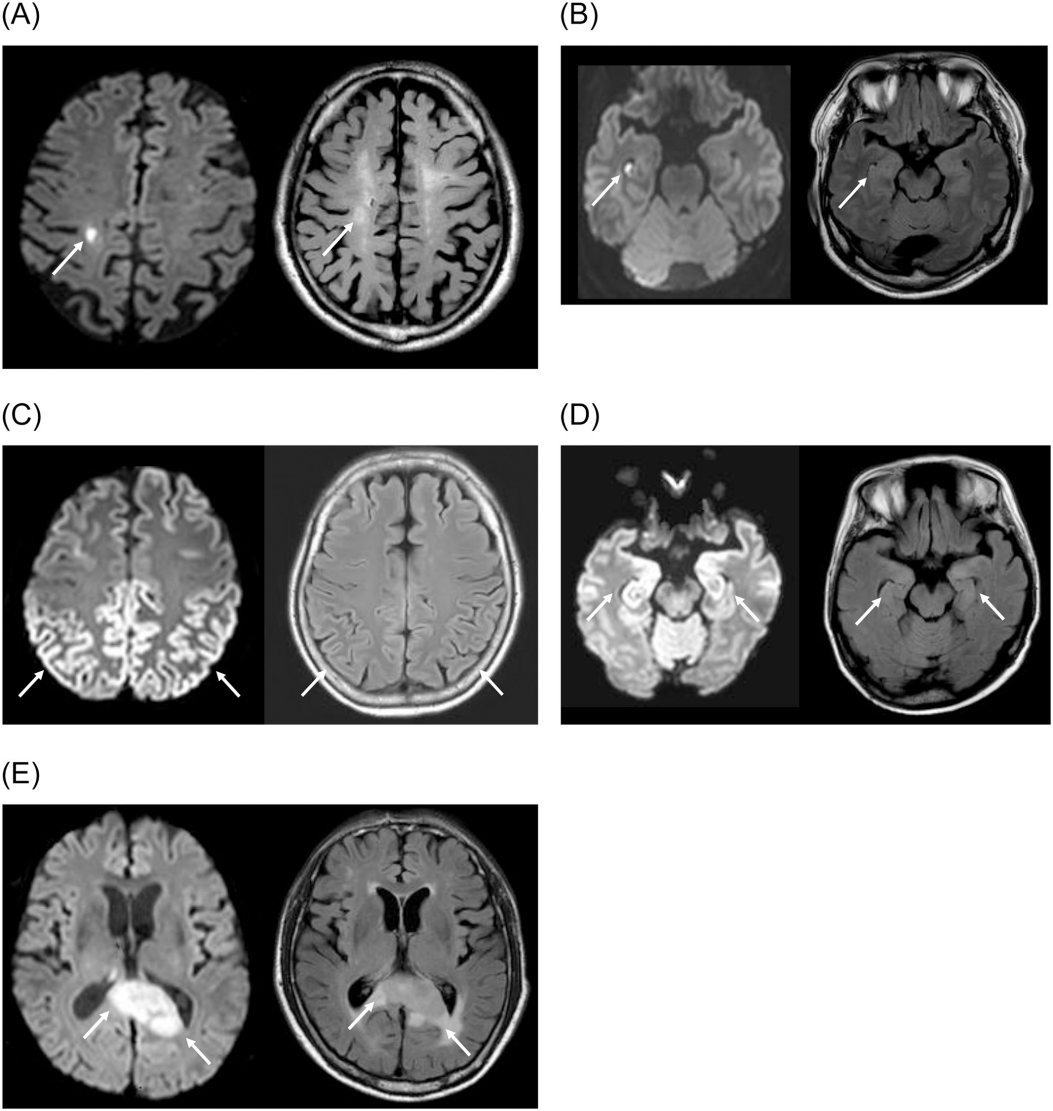
Exemplary diagnoses of recent small subcortical infarct, transient global amnesia, Creutzfeldt-Jakob disease, encephalitis and lymphoma made on diffusion-weighted imaging. (a) A 62 year old man with memory impairment since one year ago was subsequently diagnosed with vascular dementia. Axial diffusion-weighted image (left, b value = 1000 sec/mm2, slice thickness/gap, 2/0 mm) showed a tiny diffusion restricted lesion in the right paracentral lobule suggesting a recent small subcortical infarct (arrow). There was hyperintensity in the corresponding area (arrow) on the axial fluid-attenuated inversion recovery image (right), which was indistinguishable from white matter hyperintensity due to severe small vessel disease. (b) A 50 year old man with a history of transient memory impairment was subsequently diagnosed with transient global amnesia. Axial, diffusion-weighted image (left, b value = 1000 sec/mm2, slice thickness/gap, 2/0 mm) showed a tiny diffusion restricted lesion in the right hippocampus (arrow). This lesion was not demonstrable in the corresponding area (arrow) on the axial fluid-attenuated inversion recovery image (right). (c) A 63 year old woman who presented with rapidly progressive memory decline was subsequently diagnosed with Creutzfeldt-Jakob disease. Axial, diffusion-weighted image (left, b value = 1000 sec/mm2, slice thickness/gap, 2/0 mm) showed diffuse cortical diffusion restriction in bilateral parietal lobes (arrows) with subtle hyperintensity in the corresponding area (arrows) on the axial fluid-attenuated inversion recovery image (right). (d) A 50 year old woman who reported memory impairment following bone marrow transplantation for myelodysplastic syndrome was subsequently diagnosed as encephalitis on multidisciplinary consensus. Axial diffusion-weighted image (left, b value = 1000 sec/mm2, slice thickness/gap, 2/0 mm) showed diffusion restriction in bilateral medial temporal lobes (arrows), with subtle hyperintensity in the corresponding area (arrows) on the axial fluid-attenuated inversion recovery image (right). (e) A 75 year old man who presented with progressive memory impairment was subsequently diagnosed as diffuse large B cell lymphoma on stereotactic biopsy. Axial diffusion-weighted image (left, b value = 1000 sec/mm2, slice thickness/gap, 2/0 mm) showed diffusion restriction associated with a mass-like lesion involving the splenium of the corpus callosum that was hyperintense with perilesional edema on the axial fluid-attenuated inversion recovery image (right).

Imaging findings of recent small subcortical infarct, CJD, TGA, encephalitis and lymphoma on DWI and FLAIR image are shown in Fig 2. There were positive findings on DWI in the dominant hemisphere in 51% (54/106 patients).

#### Acute or subacute infarct

All recent small subcortical infarcts were not associated with signal changes on the T2-weighted image, FLAIR image or T1-weighted image except in one case in which the signal change associated with diffusion restricted lesion was indistinguishable with nonspecific white matter hyperintensities. There were 63 recent small subcortical infarcts (mean maximal diameter, 8 mm; range, 4−19 mm) in 54 patients including 9 patients with 2 recent small subcortical infarcts. There were 12 lesions (12/63; 19.0%) located in the basal ganglia or thalamus, and 41 lesions (41/63; 65.0%) located in the white matter. Among 16 lesions for which follow up MRIs were available (median interval, 631 days), 13% (2/16 lesions) of recent small subcortical infarct developed into lacune, 44% (7/16 lesions) developed into white matter hyperintensity and 44% (7/16 lesions) disappeared.

#### Creutzfeldt-Jakob disease

There were 11 patients amongst 3,298 patients (0.33%) who were diagnosed as CJD, and all 11 patients (100%) showed positive findings on DWI. All patients (11/11; 100%) had asymmetric cortical diffusion restriction, and parieto-temporal lobes were involved most consistently. In 4 patients (4/11; 36%), DWI positive lesions were not evident on the T2-weighted image, FLAIR image or T1-weighted image. All cases fulfilled the diagnostic criteria as probable sporadic CJD according to the diagnostic criteria by the Centers for Disease Control and Prevention [14].

#### Transient global amnesia

TGA appeared as single or several diffusion restricted foci in the hippocampus with subsequent improvement of memory impairment. There were 3 patients (3/5; 60%) with bilateral diffusion restricted lesions. Diffusion restricted lesions associated with TGA were not demonstrable on the T2-weighted image, FLAIR image or T1-weighted image.

#### Encephalitis

In 4 patients with encephalitis, there was diffusion restriction associated with T2/FLAIR hyperintensity which was most frequently observed in bilateral medial temporal lobes (3/4; 75%). Diffusion restriction on DWI enabled formulating differential diagnoses for the lesions which included low grade glioma, vasculitis and demyelinating lesion. There were 2 cases diagnosed as autoimmune encephalitis, 1 case as rhombencephalitis and 1 case as encephalitis of unknown cause.

#### Lymphoma

In patients with lymphoma, there was diffusion restriction associated with space-occupying lesion with perilesional edema. Relatively homogeneous diffusion restriction with low apparent diffusion coefficient aided in reaching the diagnosis of lymphoma and differential diagnoses included high grade glioma and metastasis. All cases that were pathologically confirmed were diffuse large B cell lymphoma.

### Subgroup analysis

There was a trend towards a higher diagnostic yield in patients ≥ 70 years of age (3.6%; 75/2,098) compared with patients < 70 years of age (2.6%; 31/1,200; *P* = .12). The diagnostic yield of DWI in patients with cognitive impairment was 3.7% (49/1,322) in men and 2.9% (132/57/1,976) in women. The diagnostic yield of DWI in patients with cognitive impairment by age group and sex is presented in Table 2.

**Table 2:** Diagnostic yield of diffusion-weighted imaging in patients with cognitive impairment by age groups and sex.

	Diagnostic yield, % (proportion; 95% CI)	*P* value
Overall diagnostic yield	3.2% (106/3,298; 2.6–3.9%)	
Age		.12
Age ≥ 70 years	3.6% (75/2,098; 2.8–4.5%)	
Age < 70 years	2.6% (31/1,200; 1.8–3.7%)	
Sex		.20
Male	3.7% (49/1,322; 2.8–4.9%)	
Female	2.9% (57/1,976; 2.2–3.7%)	

CI = confidence interval.

**Fig 3:**
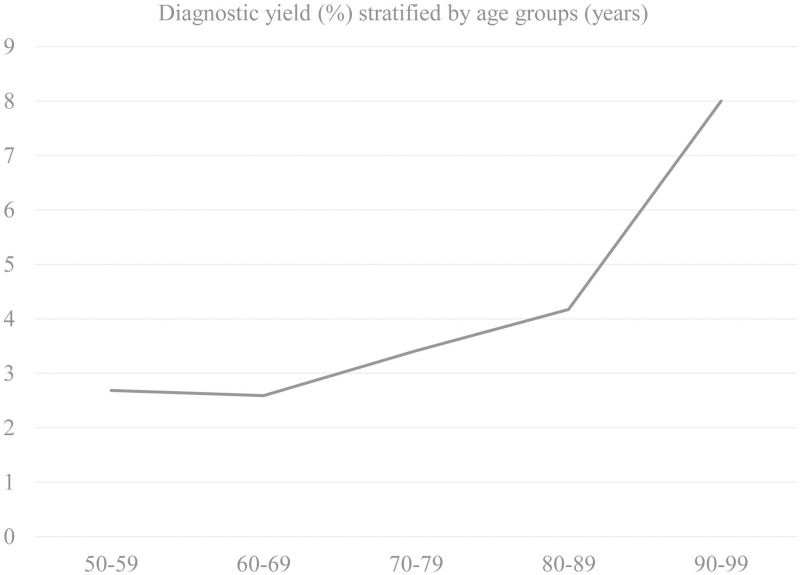


When the diagnostic yield of DWI in patients with cognitive impairment was stratified by age groups, there was an incremental increase from the group 60–69 years (2.6%; 20/773), 70–79 years (3.4%; 49/1,435), 80–89 years (4.2%; 24/575) to 90–99 years (8.0%; 2/25) (Fig 3).

### Vascular risk factors

Among 106 patients with positive findings on DWI, at least 1 vascular risk factor was found in 87% (92/106 patients), and 2 or more vascular risk factors were found in 60% (64/106 patients). In patients with acute or subacute infarct, at least 1 vascular risk factor was found in 93% (77/83 patients), and 2 or more vascular risk factors were found in 71% (59/83 patients). In patients ≥ 70 years of age, there was a trend towards a higher proportion of patients with 2 or more vascular risk factors (75%; 50/67) than in patients < 70 years of age (58%; 7/12; *P* = .23).

## Discussion

There is a paucity of evidence regarding the need and added value of DWI in the evaluation of patients with cognitive impairment. We evaluated the diagnostic yield of DWI in 8,820 consecutive patients attending outpatient clinic for cognitive impairment. The overall diagnostic yield of DWI was 3.2% (106/3,298), which included acute or subacute infarct such as recent small subcortical infarct for which the diagnostic yield was 1.6% (54/3,298). The diagnostic yield was 0.33% (11/3,298) for CJD, 0.15% (5/3,298) for TGA, 0.12% (4/3,298) for encephalitis and 0.09% (3/3,298) for lymphoma, respectively. In addition, there was a trend towards a higher diagnostic yield in the older age group with ≥ 70 years and there was an incremental increase from age 60. While the diagnostic yield of DWI in patients with cognitive impairment was low, DWI supported diagnoses of important causes of cognitive impairment with potential diagnostic or therapeutic implications.

Previous studies have reported the incidence of cerebral microinfarct in patients with cognitive impairment as 2.0% (16/783) − 3.8% (13/343) [20,21] with the highest sensitivity on DWI [22]. This study was by far the largest cohort reported to date evaluating the incidence of recent small subcortical infarct. While the pathogenesis of recent small subcortical infarct remains unclear, cerebral microinfarcts in the perfusion territory of a small artery or arteriole confirmed neuropathologically were shown to cause measurable disruption to structural brain connections [23]. This may underlie neurological dysfunction seen in vascular dementia and serve as the mechanistic link between cerebrovascular disease including small vessel disease and dementia [11,20,23]. In addition, previous studies reported variable fates of recent small subcortical infarct in keeping with the results of our study [24,25]. Diagnosing recent small subcortical infarct on DWI and estimating its overall burden are important in establishing the clinical impact of these lesions on vascular cognitive impairment [21].

While the diagnostic yield of DWI for CJD was low (0.33% [11/3,298]), CJD is an important cause of subacute cognitive impairment for which an early diagnosis is a requirement for future treatment [26–28]. The ACR Appropriateness Criteria recommends DWI to be included in patients with suspected prion disease [29], and previous studies showed the detection rate of DWI for sporadic CJD to range from 73% to 100% with a pooled detection rate of 91% in a recent meta-analysis [30–35]. A newly proposed criterion incorporating DWI findings in at least 1 positive brain region showed the superior diagnostic performance to that of standard diagnostic criteria for sporadic CJD and similar diagnostic performance to that of the cerebrospinal fluid test [33]. Moreover, increased extent and degree of DWI signal intensity were shown to correlate with disease duration of sporadic CJD and the degree of spongiosis determined histopathologically [33].

TGA is recognized as a cause of cognitive impairment for which subsequent symptomatic improvement is seen, and a transient dysfunction of medial temporal lobes is regarded as one of the main pathophysiologic mechanisms [36]. Previous studies reported variable detection rate of hippocampal diffusion restricted lesions on DWI in patients suspected of TGA from 36% to 84% with a pooled detection rate of 39% in a recent meta-analysis [36–39]. The diagnostic criteria for TGA rely heavily on the clinical history of anterograde amnesia that is witnessed [40] but studies have shown that the onset of symptoms was only witnessed in 70% (274/390) [36]. This underscores the value of positive findings on DWI in the diagnostic process of TGA, and DWI was particularly helpful in achieving the diagnosis of TGA in patients with diagnostic uncertainty [36].

Our study showed that there was a trend towards a higher diagnostic yield in the older age group with ≥ 70 years and there was an incremental increase in the overall diagnostic yield from age 60. Patients with acute or subacute infarct showed higher mean age compared with patients with TGA and CJD although the age range was also wider for patients with acute or subacute infarct. Age is a well-established risk factor for cerebrovascular disease [41,42], and vascular risk factors were more frequently observed in the older patient group with acute or subacute infarct in our study. Moreover, vascular dementia is considered to be the second most common cause of dementia after Alzheimer’s disease with a higher mortality rate [43,44], and the prevalence of vascular dementia is reported to increase linearly with age similar to Alzheimer’s disease [45,46]. This further substantiates the need for recognizing vascular dementia in the older age group by including DWI in the older age group.

The limitations of our study included its single center, retrospective nature. Diagnostic yield depends on disease prevalence, which can vary by institution and location. Reports with no positive findings on DWI were regarded as test negative having confirmed that there were no false negative cases in 200 randomly selected cases. A sample size of 200 produces a two-sided 95% CI with a width equal to 0.034 when the sample proportion is 0.990 and may be considered adequate. We did not evaluate the long-term outcome for patients with positive findings on DWI and whether positive findings on DWI would result in long term prognosis or survival benefit.

In conclusion, despite its low diagnostic yield, DWI supported diagnosis that underlie cognitive impairment including acute or subacute infarct, CJD, TGA, encephalitis and lymphoma. This study provides evidence based on a large cohort study for the need and added value of DWI that may be used for establishing and refining guidelines in the evaluation of patients with cognitive impairment.

## Supporting information

S1 File Data for patient characteristics, vascular risk factors and diagnostic yield. (PDF)

## Abbreviations

DWI: diffusion-weighted imaging
TGA: transient global amnesia
CJD: Creutzfeldt-Jakob disease
FLAIR: fluid-attenuated inversion recovery
