# Supplementary material for: Diagnostic yield of diffusion-weighted brain MR imaging in patients with cognitive impairment: Large cohort study with 3,298 patients
Source: PLoS One. 2022 Sep 22;17(9):e0274795. doi: 10.1371/journal.pone.0274795 (PMC9498979; doi:10.1371/journal.pone.0274795)
Supplement: S1 File — (PDF) [file pone.0274795.s001.pdf]

#	Se	HT	Hyp	DM	Sm	Prev	Pre	AF	Educ tion (yea	MM	CDR	Han	Dx	1: TP 2:	Late	Loc atio n	size (mm	1: re co	1: f/u MR (FLAI	Follow up interval
1	F	0	1	0	0	0	0	0	1	17	0.5	R	13	1	2	10	4	1	0	
2	F	0	0	1	0	0	0	0	16	19	1	R	11							
3	M	1	0	1	1	1	0	0	16	18	1	R	13	1	2	12	12	1	0	
4	F	1	0	0	0	0	0	1	14	28	0.5	R	13	1	1	10	7	1	0	
5	F	1	0	0	0	0	1	1	16	21	0.5		13	1	2	1	6	1	0	
6	M	0	0	0	1	0	0	0	0.5	18	0.5	R	13	1	2	3, 10	6	1	0	
7	F	0	1	0	0	0	1	0	6	23	0.5		13	1	1	3	17	1	0	
8	M	1	0	0	2	0	0	0	14	28	0.5	R	13	1	1	8	7	0	0	
10	M	1	1	1	2	1	0	0	16	28	0.5		13	1	2	3	7	1	1	295
11	M	1	0	0	1	0	0	0	16	20	1	R	19		2	1				
12	M	1	1	0	1	1	1	0	16	22	0.5	R	13	1	1	1	5	1	0	
13	M	1	0	0	2	0	0	0	16	22	0.5	R	13	1	2	10	9	1	0	
14	M	0	0	1	2	0	0	0	16	28	1	R	13	1	2	1	5	1	1	73
15	M	1	0	0	1	1	1	0	16	25	0.5	R	13	1	1	1	7	1	0	
16	F	1	0	0	1	0	0	0	0.5	15	0.5	R	13	1	3	12	19	1	0	
17	F	1	0	1	0	0	1	0	0	18	1	R	13	1	2	11	7	1	0	
18	F	1	1	0	0	1	1	1	20	23	1	R	13	1	3	1	26	3	0	
19	F	1	1	1	0	1	1	0	8	25	0.5	R	13	1	2	1	7	1	0	
22	F	0	0	1	0	0	0	0	16	12	2	R	13	1	2	1	30	3	0	
23	F	1	0	0	0	0	0	0	6	5	3	R	12	1	3	1				
24	F	1	0	0	0	0	0	0	4	20	0.5	R	13	1	1	0,11	52	3	0	
25	F	1	1	0	0	0	0	0	12	20	1	R	13	1	2	10	7	2	0	
26	F	0	1	0	0	0	0	0	6	29	0.5	R	13	1	2	1	8	1	0	
27	M	1	0	1	0	1	0	0	4	26	0.5	R	13	1	1	10	4	1	0	
28	M	0	0	0	1	0	0	0	6	19	1	R	13	1	1	10	5	1	0	
29	F	1	0	0	0	1	0	1	8	21	0.5	R	13	1	1	1	8	1	0	
33	M	0	0	0	1	0	0	0	16	24	0.5	R	18		3	1				
34	F	1	1	1	0	0	0	0	1	12	0.5	R	13	1	2	12	32	3	1	
35	M	1	1	1	0	0	0	0	5	30	0.5	R	13	1	1	11	8	1	0	
37	M	0	1	1	2	0	0	0	11	27	0.5	R	14	1	2	1				
39	M	1	0	1	1	0	0	0	16	29	0.5	R	13	1	2	10	4		1	
41	M	0	0	0	0	0	0	0	6	15	2	**R**	13	1	2	2, 10	12		0	
43	F	1	0	1	0	1	1	0	0	7	2	R	13	1	2	2,13	9		0	
45	F	1	0	0	0	0	0	0	6	23	0.5	R	17	1	1	1				
47	F	1	0	0	0	0	0	0	6	27	0.5	R	13	1	2	9	16	1	0	
48	M	1	1	0	1	0	0	0	4	22	0.5	R	13	1	1	10	5	1	2	
49	F	1	1	0	0	0	0	0	12	19	1	R	13	1	1	1	6	1	1	631
50	F	1	0	1	0	1	0	0	6	25	0.5	R	13	1	3	10	23	3	0	
51	M	1	0	0	0	0	0	0	16	20	1	R	13	1	1	9	5	1	1	
52	F	1	1	1	0	0	0	0	0	20	0.5	R	13	1	1	1	6	1	0	
53	M	0	1	0	1	0	0	0	8	21	0.5	R	13	1	2	5	7	1	0	

57	F	1	1	0	1	0	0	0	6	16	1	R	13	1	3	3,10	3	2	1	118
59	F	1	0	0	0	0	0	0	8	26	0.5	R	13	1	2	10	5	1	1	376
60	M	1	0	0	1	0	0	0	16	28	0.5	R	13	1	1	10	5	1	1	830
61	M	1	1	1	1	0	0	0	20	10	2	R	13	1	2	2,10	8	1	1	
63	M	0	0	0	0	0	0	0	0.5	15	1	R	15		3					
64	M	0	1	0	1	0	0	0	16	26	0.5	R	14	1	1					
68	F	0	0	0	0	0	0	1	7	24	0.5	R	13	1	2	1	10	1	2	84
69	F	0	1	0	0	1	1	0	0	15	0.5	R	13	1	1	8	10	0	0	
70	F	1	1	0	0	0	0	1	6	21	0.5	R	13	1	2	3, 10	5	1	0	
71	F	1	0	0	0	1	0	1	0.5	18	0.5	R	13	1	1	2, 3	22	3	1	
72	M	1	1	1	1	0	0	1	10	25	0.5	R	13	1	1	12	7		1	
73	F	1	1	0	0	0	0	0	1	20	0.5	R	13	1	3	13	7	1	1	
74	F	1	0	0	0	1	0	0	0	20	0.5	R	13	1	1	12	4	1	1	909
